# Inability to get needed health care during the COVID-19 pandemic among a nationally representative, diverse population of U.S. adults with and without chronic conditions

**DOI:** 10.1186/s12889-023-16746-w

**Published:** 2023-09-26

**Authors:** Stephanie A. Ponce, Miciah Wilkerson, Randy Le, Anna María Nápoles, Paula D. Strassle

**Affiliations:** Division of Intramural Research, National Institute on Minority Health and Health Disparities, National Institutes of Health, Bethesda, MD USA

**Keywords:** COVID-19, Chronic diseases, Unmet health care, Delayed medical care, Disparities

## Abstract

**Background:**

Delays in health care have been observed in the U.S. during the COVID-19 pandemic; however, the prevalence of inability to get needed care and potential disparities in health care access have yet to be assessed.

**Methods:**

We conducted a nationally representative, online survey of 5,500 American Indian/Alaska Native, Asian, Black/African American, Latino (English- and Spanish-speaking), Native Hawaiian/Pacific Islander, White, and multiracial adults between 12/2020–2/2021 (baseline) and 8/16/2021–9/9/2021 (6-month follow-up). Participants were asked “Since the start of the pandemic, was there any time when you did not get medical care that you needed?” Those who responded “Yes” were asked about the type of care and the reason for not receiving care. Poisson regression was used to estimate the association between sociodemographics and inability to receive needed care; all analyses were stratified by chronic condition status. Chronic conditions included: chronic obstructive pulmonary disease (COPD), heart conditions, type 2 diabetes, chronic kidney disease or on dialysis, sickle cell disease, cancer, and immunocompromised state (weakened immune system).

**Results:**

Overall, 20.0% of participants at baseline and 22.7% at follow-up reported not getting needed care. The most common reasons for being unable to get needed care included fear of COVID-19 (baseline: 44.1%; follow-up: 47.2%) and doctors canceled appointment (baseline: 25.3%; follow-up: 14.1%). Routine care (baseline: 59.9%; follow-up: 62.6%) and chronic care management (baseline: 31.5%; follow-up: 30.1%) were the most often reported types of delayed care. Fair/poor self-reported physical health was significantly associated with being unable to get needed care despite chronic condition status (≥ 1 chronic condition: aPR = 1.36, 95%CI = 1.04–1.78); no chronic conditions: aPR = 1.52, 95% CI = 1.28–1.80). The likelihood of inability to get needed care differed in some instances by race/ethnicity, age, and insurance status. For example, uninsured adults were more likely to not get needed care (≥ 1 chronic condition: aPR = 1.76, 95%CI = 1.17–2.66); no chronic conditions: aPR = 1.25, 95% CI = 1.00–1.56).

**Conclusions:**

Overall, about one fifth of participants reported being unable to receive needed care at baseline and follow-up. Delays in receiving needed medical care may exacerbate existing conditions and perpetuate existing health disparities among vulnerable populations who were more likely to have not received needed health care during the pandemic.

**Supplementary Information:**

The online version contains supplementary material available at 10.1186/s12889-023-16746-w.

## Background

In response to the massive demands on the healthcare system and strained medical supply chains caused by the COVID-19 pandemic in the United States (U.S.), in March 2020 the Centers for Medicare and Medicaid Services (CMS) recommended to prioritize essential health care services and delay or halt those that were deemed non-emergency or unessential [1]. In accordance with these recommendations, many U.S. states issued mandates or formal recommendations limiting non-essential care, such as elective surgeries and routine dental and eye care to ensure resources were focused on the treatment and testing of COVID-19 [1]. By June 30, 2020, the Centers of Disease Control and Prevention (CDC) estimated that 41% of U.S. adults had delayed or avoided medical care because of concerns related to COVID-19 [2]. In April 2020, the CMS released guidelines to continue elective surgeries and procedures in U.S. areas with low and stable incidence of COVID-19 cases [3]. However, in September 2020 more than one in three U.S. adults were still reporting forgoing health care because of concerns of possible COVID-19 exposure or continued limited services [4]. Similarly, other studies conducted in the U.S. have found that rates of elective surgeries, dental care, treatment of heart attack and stroke, gender affirming care, visits for chronic disease management, and cancer screenings substantially declined during the first year of the pandemic [5–7]. Decreased rates in U.S.-based emergency hospitalizations, emergency department visits, asthma emergencies, and inpatient clinic visits of chronic diseases have also been documented, none of which should have been impacted by the pandemic [2, 8–12].

Chronic diseases such as cancer, diabetes, and heart disease are among the top health problems in the U.S [13], and having one or more chronic conditions has been associated with decreased quality of life, longer hospital stays, higher mortality, and overall poorer health outcomes, especially when faced with infectious diseases such as COVID-19 [14, 15]. Patients with chronic conditions require medical support to develop a disease management plan which often requires regular follow-up care [16]. Disrupted access to health care for management of chronic diseases and routine care has been shown to result in preventable acute service, reduced quality of life, and life-threatening progression of disease [17, 18]. Similarly, other vulnerable populations such as transgender and non-binary identifying individuals, older individuals, and individuals with unmet health-related needs also require more frequent health care interactions [19, 20]. Given the potentially detrimental impact of not getting needed health care, it is essential to assess how often adults in the U.S. experienced being unable to get needed care, why they were unable to get care, and the types of care that weren’t received, as well as identify communities at highest risk for not having received needed care.

Thus, the purpose of this study was to estimate the prevalence of being unable to get needed health care during the COVID-19 pandemic among U.S. adults with and without chronic conditions (overall and by type of care), identify the major reasons why individuals were unable to get health care, and assess sociodemographics that were associated with being more likely to not having received needed care using a nationally representative survey of American Indian/Alaska Native, Asian, Black/African American, Latino, Native Hawaiian/Pacific Islander, White, and multiracial adults living in the U.S. We hypothesized there would be substantial racial-ethnic and sociodemographic disparities in health care delays and that all types of health care would be impacted.

## Methods

### Study population and survey development

We used data from the COVID-19’s Unequal Racial Burden (CURB) online survey, which was administered by YouGov. Panel members (~ 1.8 million) were recruited through a variety of methods to ensure diversity, and then proximity matched to a theoretical target sample drawn from the 2018 American Community Survey 1-year sample data. The target sample for the CURB survey included 1,000 Asian, Black/African American, Latino (including 500 Spanish-speaking), and White adults, and 500 American Indian/Alaska Native, Native Hawaiian/Pacific Islander, and multiracial adults living in the U.S. (*n* = 5,500 total). Matched panel members who completed the online survey were then weighed to obtain a nationally representative sample within each racial-ethnic group (e.g., Asian participants were weighted to represent all Asians living in the United States). Details about the CURB survey development, sampling design, and participant demographics have been published previously [21, 22]. The CURB survey was first created in English, translated into Spanish by an American Translators Association certified translator, and then finalized by four bilingual/bicultural researchers via reconciliation and decentering methods.

Baseline surveys were completed between December 8, 2020 and February 17, 2021 (20.0% initial response rate). The 6-month follow-up survey was completed between August 16, 2021 and September 9, 2021 (35.1% response rate). The National Institutes of Health Office of Institutional Review Board Operations determined that this study does not qualify as human subjects research because data were de-identified (IRB# 000166).

### Inability to get needed health care during the COVID-19 pandemic

At baseline, all participants were asked “Since the start of the pandemic, was there any time when you did not get medical care that you needed?” Those who responded “Yes” were additionally asked “What are the reasons you have put off getting the medical care that you needed?” and “What type of medical care did you put off?” The reasons for putting off care included: 1) No health insurance even before the pandemic; 2) Loss of health insurance since the pandemic; 3) Fear of getting COVID-19 by visiting the doctor; 4) Not enough money to pay for the visit or co-pays; 5) Doctor canceled appointments; 6) Doctor discouraged new appointments; 7) No time/too busy; 8) Other, which included an open-ended, write-in response. Participants were able to select multiple reasons. The types of medical care included: 1) Follow-up of a chronic condition (For example: diabetes, hypertension, cancer); 2) Treatment of an urgent condition (For example: fall, heart pain); 3) Routine care or checkup; 4) Cancer screening; 5) Medical care for possible COVID-19; and 6) Other, which included an open-ended, write-in response. Participants were again allowed to select multiple responses. At the 6-month follow-up, the same set of questions were asked about health care and delayed health care in the past 6 months (i.e., since the time of the last survey). At follow-up, medical care for possible COVID-19 was not included as a possible response option, and instead captured that information elsewhere in the survey (data not included in analysis).

### Race-ethnicity and other sociodemographics

At baseline, all participants were asked “Which one of the following would you say best represents your race-ethnicity?” with response options of Latino/a/x or Hispanic, American Indian or Alaska Native, Asian, Black or African American, Pacific Islander, White, or multiracial. Among Asian participants, we also asked to provide national origin (Asian Indian, Chinese, Filipino, Japanese, Korean, Vietnamese, and other Asian). Participants who identified as Pacific Islander were additionally asked whether they identified as Native Hawaiian, Guamanian or Chamorro, Samoan, or other Pacific Islander. Latino participants were asked about national origin (Mexican/Mexican American/Chicano, Puerto Rican, Cuban/Dominican, Central American, South American, and other Hispanic/Latino/Spanish) and also stratified into English- and Spanish-speaking based on survey language preference; 87.6% of Latino participants who took the survey in Spanish also self-reported poor English-speaking skills (compared to only 12.4% of Latino participants who took the survey in English).

At baseline, participants were also asked “Has a medical doctor ever told you that you have any of the following conditions?” Chronic conditions included: chronic obstructive pulmonary disease (COPD), heart conditions (such as heart failure, coronary artery disease, or cardiomyopathies), type 2 diabetes, chronic kidney disease or on dialysis, sickle cell disease, cancer within the past year, immunocompromised state (weakened immune system) from solid organ transplant, obesity, COVID-19, and none of the above. Participants were able to check multiple conditions. Individuals who selected any of the above conditions besides obesity and COVID-19 were considered to have a chronic condition.

### Statistical analyses

Descriptive statistics were used to estimate the proportion of adults who were unable to get needed care, the reasons for not getting care, and the types of care during the COVID-19 pandemic, overall and stratified by race-ethnicity and chronic condition status, at both baseline and 6-month follow-up. Multivariable Poisson regression was used to estimate the independent associations between race-ethnicity, other sociodemographic characteristics, and the inability to seek needed care during the pandemic. Separate models were run for participants with and without chronic conditions, as we expected individuals with at least one chronic condition to have different health care seeking behaviors and needs, compared to those without chronic conditions [23]. All models included race-ethnicity, age (18–29, 30–39, 40–49, 50–59, 60–69, ≥ 70), gender (male, female, transgender or non-binary), self-reported physical health (excellent/very good/good, fair/poor), insurance status (any private, public only, uninsured), educational attainment (less than high school, high school/Tests of General Educational Development (GED), some college/vocational school, college degree or higher), family annual income (< $20,000, $20,000-$59,999, $60,000-$99,999, ≥ $100,000), U.S. Census division (New England, Middle Atlantic, East North Central, West North Central, South Atlantic, East South Central, West South Central, Mountain, Pacific), and urbanicity (big city, smaller city, suburban area, small town, rural). Limited English proficiency was defined as being able to speak English “not at all,” “poorly,” or “fairly well.” Missingness was minimal for all variables except for family annual income (*n* = 659 [unweighted] selected “Prefer not to say”); these individuals were not included in our multivariable models.

All analyses were performed using SAS version 9.4 (SAS Inc., Cary, NC). All baseline analyses were weighted to produce nationally representative estimates within each racial-ethnic group and counts were rounded for interpretation. Due to the relatively low response rate, follow-up results were unweighted in all analyses.

## Results

**Fig. 1:**
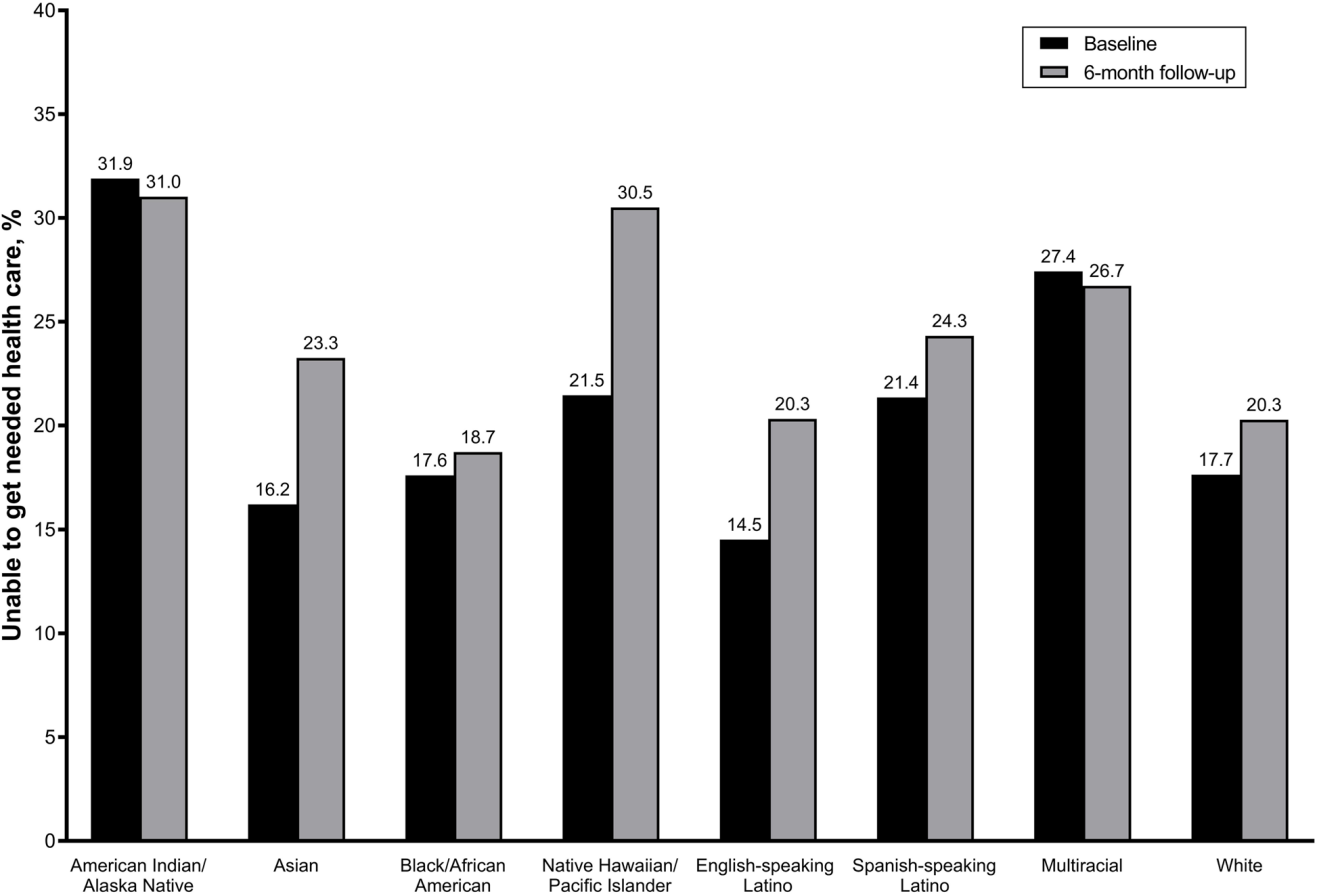
Prevalence of being unable to get needed care reported at baseline and 6-month follow-up, stratified by race/ethnicity. Baseline estimates were weighted to be nationally representative within each racial/ethnic group

Overall, 20.0% of participants reported being unable to receive needed care at baseline and 22.7% reported being unable to receive needed care at 6-month follow-up. Among those who reported being unable to receive care at baseline, 24.9% also reported being unable to receive care again at follow-up. The prevalence of being unable to receive needed care was highest among American Indian/Alaska Native (baseline: 31.9%; follow-up: 31.0%), followed by multiracial (baseline: 27.4%; follow-up: 26.7%) and Native Hawaiian/Pacific Islander (baseline: 21.5%; follow-up: 30.5%) adults, Fig. 1. When stratified by chronic condition status, almost half (48.1%) of American Indian/Alaska Native adults with at least one chronic condition reported being unable to get needed care at baseline, compared to 24.2% of American Indian/Alaska Native without a chronic condition, Supplemental Fig. 1A. Additionally, English-speaking Latino (24.3%) and multiracial adults (36.4%) with at least one chronic condition reported a higher prevalence of being unable to get needed care, compared to their non-chronic condition counterparts at baseline.

Differences were also seen between Asian, Latino, and Native Hawaiian/Pacific Islander subgroups at baseline. Notably, among those with chronic conditions, Korean adults had a higher prevalence than the other Asian groups (31.9%); Cuban/Dominican Republic adults had a higher prevalence than the other Latino groups (43.6%); and Other Pacific Islander (35.0%) adults reported a higher prevalence of being unable to get needed care than Native Hawaiian/Pacific Islander adults, Table 1. Similar trends in the inability to get needed care across race-ethnicity and chronic condition status were seen at follow-up, Supplemental Fig. 1B.

**Table 1:** Prevalence of inability to get needed care across sociodemographics, stratified by chronic condition status, at baseline and follow-up. Baseline results weighted to be nationally representative within each racial/ethnic group

	Baseline		6-Month Follow-up	
	**Chronic condition(s)** **N (%)**	**No chronic conditions** **N (%)**	**Chronic condition(s)** **N (%)**	**No chronic conditions** **N (%)**
**Overall**	303 (26.3)	796 (18.3)	106 (24.9)	333 (22.2)
**Race/ethnicity**				
American Indian/Alaska Native	77 (48.1)	82 (24.2)	24 (42.9)	30 (25.4)
Asian	35 (19.5)	127 (15.5)	17 (23.9)	83 (23.2)
Asian Indian	10 (33.5)	28 (19.0)	1 (12.5)	19 (31.1)
Chinese	4 (11.6)	30 (12.7)	5 (27.8)	19 (16.8)
Filipino	7 (17.0)	23 (19.8)	3 (23.1)	12 (23.5)
Japanese	4 (10.9)	15 (15.2)	3 (23.1)	13 (28.9)
Korean	6 (31.9)	10 (16.8)	2 (25.0)	7 (25.0)
Vietnamese	1 (8.9)	7 (14.1)	0 (0.0)	7 (28.0)
Other Asian	4 (27.5)	14 (12.7)	3 (42.9)	6 (17.1)
Black/African American	46 (20.4)	130 (16.8)	13 (17.1)	44 (18.6)
Latino	40 (22.3)	140 (17.0)	14 (25.0	42 (20.5)
*English-speaking*^*a*^	25 (24.3)	47 (12.0)	10 (23.3)	28 (19.4)
*Spanish-speaking*^*a*^	15 (19.5)	93 (21.7)	4 (30.8)	14 (23.0)
Mexican/Mexican American/Chicano	26 (26.9)	83 (19.3)	7 (22.6)	26 (23.2)
Puerto Rican	4 (11.1)	13 (16.6)	3 (25.0)	6 (20.0)
Cuban/Dominican Republic	3 (43.6)	3 (5.2)	2 (50.0)	1 (8.3)
Central American	3 (54.9)	12(16.6)	2 (100.0)	3 (27.3)
South American	1 (12.8)	11 (11.8)	0 (0.0)	5 (19.2)
Other Hispanic/Latino/Spanish	2 (8.9)	12 (15.7)	0 (0.0)	1 (9.1)
Native Hawaiian/Pacific Islander	32 (27.0)	76 (19.8)	6 (35.3)	12 (28.6)
Native Hawaiian	14 (20.8)	37 (17.6)	4 (40.0)	5 (20.8)
Other Pacific Islander^b^	18 (35.0)	39 (22.4)	2 (28.6)	7 (38.9)
Multiracial	29 (36.4)	109 (25.8)	11 (28.2)	43 (26.4)
White	45 (20.9)	132 (16.8)	21 (18.9)	79 (20.7)
**Age group**				
18–29	41 (40.4)	234 (18.0)	9 (56.3)	51 (29.8)
30–39	36 (31.2)	212 (21.5)	11 (36.7)	77 (23.8)
40–49	50 (31.6)	158 (21.2)	15 (30.0)	66 (22.8)
50–59	77 (30.4)	107 (17.5)	28 (22.4)	83 (21.6)
60–69	59 (19.0)	62 (12.7)	36 (24.7)	47 (17.6)
≥ 70	40 (18.6)	23 (10.4)	7 (11.9)	9 (13.2)
**Gender**				
Female	133 (23.3)	411 (18.7)	58 (28.0)	196 (24.0)
Male	155 (28.1)	348 (17.1)	45 (21.0)	123 (18.5)
Transgender or non-binary^c^	14 (62.3)	36 (32.6)	3 (75.0)	14 (63.6)
**Self-reported physical health**				
Excellent/very good/good	116 (21.3)	568 (16.4)	39 (17.7)	246 (20.1)
Fair/poor	187 (30.7)	228 (26.0)	67 (32.5)	87 (31.4)
**Health insurance**				
Any private insurance	95 (20.0)	322 (16.9)	45 (23.6)	187 (22.0)
Public insurance only	150 (27.0)	265 (19.0)	50 (24.0)	84 (20.1)
Uninsured	57 (48.6)	204 (20.0)	10 (40.0)	59 (25.9)
**Primary care provider**				
Yes	235 (23.4)	527 (19.5)	95 (24.1)	214 (20.3)
No	68 (45.5)	268 (16.3)	11 (35.5)	119 (26.4)
**Immigration status**				
US-born citizen	255 (26.8)	615 (18.5)	87 (24.5)	258 (21.9)
Foreign-born citizen/legal resident	39 (22.4)	137 (17.8)	17 (26.2)	63 (22.5)
Undocumented	8 (30.0)	43 (17.3)	2 (33.3)	12 (27.9)
**English proficiency**^**d**^				
Limited	51 (42.1)	114 (23.0)	11 (37.9)	19 (19.6)
Not limited	251 (24.4)	681 (17.7)	95 (23.9)	314 (22.3)
**Highest education level**				
Less than high school graduate	43 (34.9)	77 (20.4)	4 (18.2)	15 (30.0)
High school/GED	108 (26.3)	229 (16.6)	18 (17.6)	54 (17.0)
Some college/vocational school	88 (24.3)	246 (18.6)	51 (32.1)	100 (21.9)
College degree or higher^**e**^	63 (24.9)	243 (19.3)	33 (23.1)	164 (24.1)
**Family annual income**^**f**^				
< $20,000	77 (29.9)	171 (20.4)	21 (28.8)	41 (22.0)
$20,000-$59,999	121 (29.0)	292 (19.4)	42 (28.8)	119 (23.9)
$60,000-$99,999	46 (22.2)	148 (19.3)	19 (23.8)	76 (23.8)
≥ $100,000	32 (20.3)	94 (14.2)	15 (19.2)	63 (20.3)
Prefer not to say	27 (23.9)	92 (15.9)	9 (18.4)	34 (18.0)
**Census division**				
New England	6 (21.7)	27 (20.3)	9 (37.5)	8 (16.7)
Middle Atlantic	28 (22.2)	93 (18.7)	14 (25.0)	42 (20.9)
East North Central	27 (22.8)	83 (19.0)	10 (21.3)	42 (23.0)
West North Central	16 (30.9)	32 (18.8)	8 (29.6)	14 (23.3)
South Atlantic	56 (24.0)	141 (16.9)	12 (14.1)	62 (22.5)
East South Central	17 (26.3)	37 (19.8)	6 (35.3)	16 (25.8)
West South Central	33 (25.4)	98 (18.2)	8 (20.0)	48 (25.8)
Mountain	39 (33.4)	76 (17.3)	8 (25.0)	27 (20.1)
Pacific	81 (28.3)	208 (18.8)	31 (31.6)	74 (20.9)
**Residence urbanicity**				
Big city	82 (28.1)	221 (19.3)	26 (25.0)	86 (24.8)
Smaller city	57 (27.7)	160 (19.6)	17 (23.6)	47 (20.0)
Suburban area	74 (22.0)	227 (17.1)	33 (27.3)	127 (22.5)
Small town	34 (25.2)	89 (17.2)	15 (27.3)	28 (18.5)
Rural	46 (37.7)	86 (19.3)	12 (23.5)	34 (21.1)

^a^ Latino participants were stratified based on survey language preference

^b^ Other Native Hawaiian/Pacific Islander includes Guamanian or Chamorro and Samoan respondents in addition to self-reported “other” (due to small sample sizes)

^c^ Nonbinary includes individuals who reported being nonbinary, gender fluid, gender queer, 'other', and no gender

^d^ Limited English proficiency was defined as speaking English "not at all", "poorly", and "fairly well"

^e^ Includes bachelor's degree, master's degree, and doctoral or postgraduate education

^f^ Collected by YouGov at enrollment into panel and updated every 6 months

Nearly half of uninsured participants with one or more chronic conditions reported being unable to get needed care at baseline (48.6%) and follow-up (40.0%), Table 1. Compared to those who identified as male and female, transgender and nonbinary individuals also consistently reported a higher prevalence of being unable to get needed care at both baseline (chronic conditions: 62.3%; no chronic conditions: 32.6%) and follow-up (chronic conditions: 75.0%; no chronic conditions: 63.6%) regardless of chronic condition status. Similarly, individuals with fair/poor self-reported physical health reported a consistently higher prevalence of inability to get needed care (26.0%-32.5%), compared to those reporting excellent/very good/good physical health (16.4%-21.3%), Table 1. Compared to older adults with chronic conditions, adults aged 18–29 with one or more chronic conditions reported a higher prevalence of inability to get needed care (baseline: 40.4%, follow-up: 56.3%).

Adjusted associations between race-ethnicity and other sociodemographics, and inability to get needed care are reported in Table 2. At baseline, among participants without chronic conditions, American Indian/Alaska Native (adjusted prevalence ratio [aPR] = 1.31, 95% CI = 0.97–1.78) and multiracial (aPR = 1.29, 95% CI = 0.97–1.72) adults appeared more likely to not get needed care compared to White adults but estimates were imprecise, Table 2. Younger adults (e.g., 18–29 vs. 60–69 years old: aPR = 1.57, 95% CI = 1.13–2.17), those who reported fair/poor self-reported physical health (aPR = 1.52, 95% CI = 1.28–1.80), and those who had a primary care provider (aPR = 1.38, 95% CI = 1.16–1.64) were also more likely to report being unable to get needed care. Among participants with one or more chronic conditions, American Indian/Alaska Native (aPR = 1.68, 95%CI = 1.07–2.64) adults, those who were uninsured (aPR = 1.76, 95% CI = 1.17–2.66), aged 50–59 (aPR = 1.48, 95% CI = 1.02–2.16), and those who reported fair/poor self-reported physical health (aPR = 1.36, 95% CI = 1.04–1.78) were also more likely to experience inability to get needed care, Table 2.

**Table 2:** Adjusted associations between race-ethnicity, sociodemographics and inability to get needed care, stratified by chronic condition status, at baseline and follow-up. Baseline results weighted to be nationally representative within each racial/ethnic group

	Baseline		6-Month Follow-up	
	**Chronic condition(s)** **aPR (95% CI)**^**a**^	**No chronic conditions** **aPR (95% CI)**^**a**^	**Chronic condition(s)** **aPR (95% CI)**^**a**^	**No chronic conditions** **aPR (95% CI)**^**a**^
**Race/ethnicity**				
American Indian/Alaska Native	1.68 (1.07, 2.64)	1.31 (0.97, 1.78)	1.57 (0.76, 3.24)	1.33 (0.84, 2.10)
Asian	0.80 (0.48, 1.35)	0.78 (0.59, 1.04)	0.77 (0.35, 1.71)	1.06 (0.73, 1.55)
Black/African American	0.79 (0.48, 1.30)	0.90 (0.68, 1.19)	0.69 (0.31, 1.56)	0.89 (0.59, 1.36)
Latino				
English-speaking	0.99 (0.56, 1.72)	0.70 (0.49, 1.00)	0.85 (0.35, 2.09)	0.86 (0.53, 1.39)
Spanish-speaking	0.57 (0.29, 1.11)	1.15 (0.83, 1.59)	1.01 (0.25, 4.00)	0.99 (0.50, 1.96)
Native Hawaiian/Pacific Islander	0.97 (0.56, 1.68)	1.10 (0.80, 1.52)	1.20 (0.39, 3.66)	1.41 (0.72, 2.76)
Multiracial	1.37 (0.80, 2.36)	1.29 (0.97, 1.72)	1.04 (0.44, 2.48)	1.13 (0.74, 1.74)
White	1.00 (ref)	1.00 (ref)	1.00 (ref)	1.00 (ref)
**Age group**				
18–29	1.53 (0.97, 2.41)	1.57 (1.13, 2.17)	1.95 (0.72, 5.29)	1.75 (1.08, 2.83)
30–39	1.45 (0.93, 2.27)	1.82 (1.32, 2.52)	1.26 (0.57, 2.77)	1.40 (0.90, 2.17)
40–49	1.41 (0.94, 2.12)	1.72 (1.23, 2.41)	1.13 (0.56, 2.29)	1.53 (0.98, 2.38)
50–59	1.48 (1.02, 2.16)	1.48 (1.04, 2.10)	0.79 (0.46, 1.36)	1.58 (1.04, 2.41)
60–69	1.00 (ref)	1.00 (ref)	1.00 (ref)	1.00 (ref)
≥ 70	0.79 (0.49, 1.29)	0.84 (0.48, 1.48)	0.31 (0.09, 1.05)	1.10 (0.48, 2.55)
**Gender**				
Female	0.85 (0.65, 1.10)	1.01 (0.87, 1.19)	1.42 (0.89, 2.25)	1.23 (0.96, 1.57)
Male	1.00 (ref)	1.00 (ref)	1.00 (ref)	1.00 (ref)
Transgender or non-binary^b^	1.61 (0.86, 3.02)	1.45 (0.98, 2.15)	1.09 (0.22, 5.30)	3.49 (1.88, 6.49)
**Fair/poor physical health**	1.36 (1.04, 1.78)	1.52 (1.28, 1.80)	1.82 (1.13, 2.92)	1.70 (1.30, 2.24)
**Health insurance**				
Any private insurance	1.00 (ref)	1.00 (ref)	1.00 (ref)	1.00 (ref)
Public insurance only	1.33 (0.97, 1.82)	1.05 (0.85, 1.28)	0.86 (0.50, 1.49)	0.92 (0.66, 1.27)
Uninsured	1.76 (1.17, 2.66)	1.25 (1.00, 1.56)	1.26 (0.55, 2.89)	1.08 (0.75, 1.56)
**Has primary care provider**	0.73 (0.52, 1.03)	1.38 (1.16, 1.64)	1.37 (0.57, 3.33)	0.82 (0.63, 1.06)
**Highest education level**				
Less than high school graduate	0.93 (0.59, 1.47)	0.72 (0.52, 1.00)	0.64 (0.21, 1.98)	1.24 (0.65, 2.34)
High school/GED	0.88 (0.61, 1.29)	0.71 (0.57, 0.90)	0.78 (0.39, 1.56)	0.60 (0.41, 0.88)
Some college/vocational school	0.82 (0.56, 1.20)	0.82 (0.67, 1.01)	1.30 (0.74, 2.29)	0.75 (0.55, 1.02)
College degree or higher^c^	1.00 (ref)	1.00 (ref)	1.00 (ref)	1.00 (ref)
**Family annual income**^**d**^				
< $20,000	1.12 (0.67, 1.88)	1.32 (0.98, 1.79)	1.18 (0.50, 2.81)	0.98 (0.61, 1.59)
$20,000-$59,999	1.20 (0.75, 1.91)	1.37 (1.05, 1.77)	1.31 (0.66, 2.61)	1.26 (0.88, 1.80)
$60,000-$99,999	1.16 (0.71, 1.92)	1.28 (0.98, 1.67)	1.25 (0.61, 2.55)	1.19 (0.83, 1.68)
≥ $100,000	1.00 (ref)	1.00 (ref)	1.00 (ref)	1.00 (ref)
**Census division**				
New England	0.67 (0.26, 1.72)	1.14 (0.72, 1.80)	0.94 (0.35, 2.57)	0.83 (0.34, 2.02)
Middle Atlantic	1.00 (ref)	1.00 (ref)	1.00 (ref)	1.00 (ref)
East North Central	0.96 (0.54, 1.72)	0.83 (0.59, 1.18)	0.77 (0.31, 1.91)	1.41 (0.87, 2.27)
West North Central	0.99 (0.50, 1.95)	0.89 (0.57, 1.39)	0.76 (0.26, 2.25)	1.23 (0.63, 2.39)
South Atlantic	0.86 (0.51, 1.44)	0.91 (0.68, 1.21)	0.67 (0.28, 1.61)	1.19 (0.76, 1.86)
East South Central	0.70 (0.35, 1.38)	1.06 (0.69, 1.62)	0.93 (0.30, 2.83)	1.22 (0.60, 2.48)
West South Central	0.89 (0.52, 1.52)	0.89 (0.65, 1.22)	0.72 (0.28, 1.86)	1.28 (0.80, 2.05)
Mountain	0.90 (0.51, 1.59)	0.82 (0.59, 1.15)	0.95 (0.35, 2.60)	0.98 (0.57, 1.67)
Pacific	0.98 (0.60, 1.60)	0.93 (0.70, 1.23)	1.23 (0.57, 2.66)	1.04 (0.68, 1.61)
**Residence urbanicity**				
Big city	1.17 (0.82, 1.65)	1.10 (0.89, 1.35)	0.78 (0.43, 1.42)	1.12 (0.83, 1.52)
Smaller city	1.10 (0.76, 1.61)	1.06 (0.85, 1.33)	0.64 (0.33, 1.25)	0.95 (0.66, 1.36)
Suburban area	1.00 (ref)	1.00 (ref)	1.00 (ref)	1.00 (ref)
Small town	0.91 (0.58, 1.42)	1.03 (0.79, 1.34)	0.88 (0.43, 1.81)	0.92 (0.58, 1.45)
Rural	1.32 (0.87, 2.01)	1.08 (0.82, 1.43)	0.60 (0.27, 1.35)	1.00 (0.65, 1.53)

^a^ Adjusted model included race-ethnicity, age, gender, English proficiency, self-reported physical health, insurance status, educational attainment, family annual income, Census division, and urbanicity

^b^ Nonbinary includes individuals who reported being nonbinary, gender fluid, gender queer, 'other', and no gender

^c^ Includes bachelor's degree, master's degree, and doctoral or postgraduate education

^d^ Collected by YouGov at enrollment into panel and updated every 6 months

Similar associations between race-ethnicity, other sociodemographics, and inability to get needed care (among those with and without chronic conditions) were seen at follow-up, although estimates were imprecise due to the smaller sample size, Table 2.

**Fig. 2:**
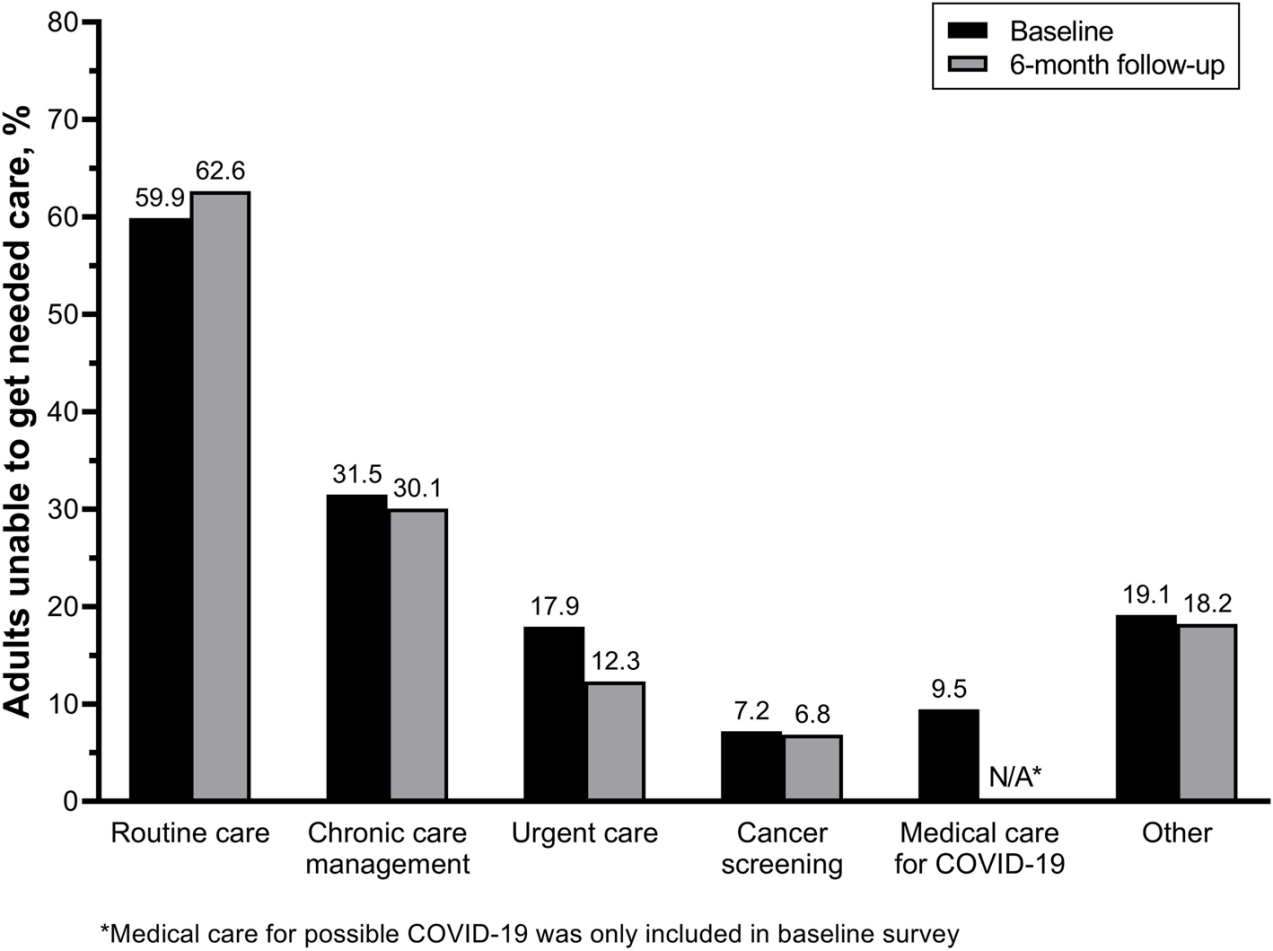
Types of care among those who reported being unable to get needed care at baseline and 6-month follow-up. Baseline estimates were weighted to be nationally representative within each racial/ethnic group

Among participants who reported being unable to get needed care, the most common types of care reported were routine care (baseline: 59.9%; follow-up: 62.6%), chronic care management (baseline: 31.5%; follow-up: 30.1%), urgent care (baseline: 17.9%; follow-up: 12.3%), and other types of care (baseline: 19.1%; follow-up: 18.2%), Fig. 2. At baseline, multiracial adults with one or more chronic condition reported the highest prevalence of inability to get chronic care management (65.9%) and routine care (72.2%); followed by English-speaking Latino (chronic care management: 64.5%; routine care: 52.4%) and American Indian/Alaska Native adults (chronic care management: 50.0%; routine care: 57.4%), Supplemental Fig. 2. Among adults with no chronic conditions, not getting needed routine care was most common among Spanish-speaking Latino (74.5%), English-speaking Latino (70.8%), and Asian (68.2%) adults.

Similar trends at 6-month follow-up were seen. Notably, over one-third of American Indian/Alaska Native adults with one or more chronic conditions (33.3%) and without chronic conditions (43.3%) reported being unable to get urgent care, which was much higher than baseline estimates (21.4% and 20.6%, respectively) and the prevalence among other racial-ethnic groups at follow-up, Supplemental Fig. 3.

**Fig. 3:**
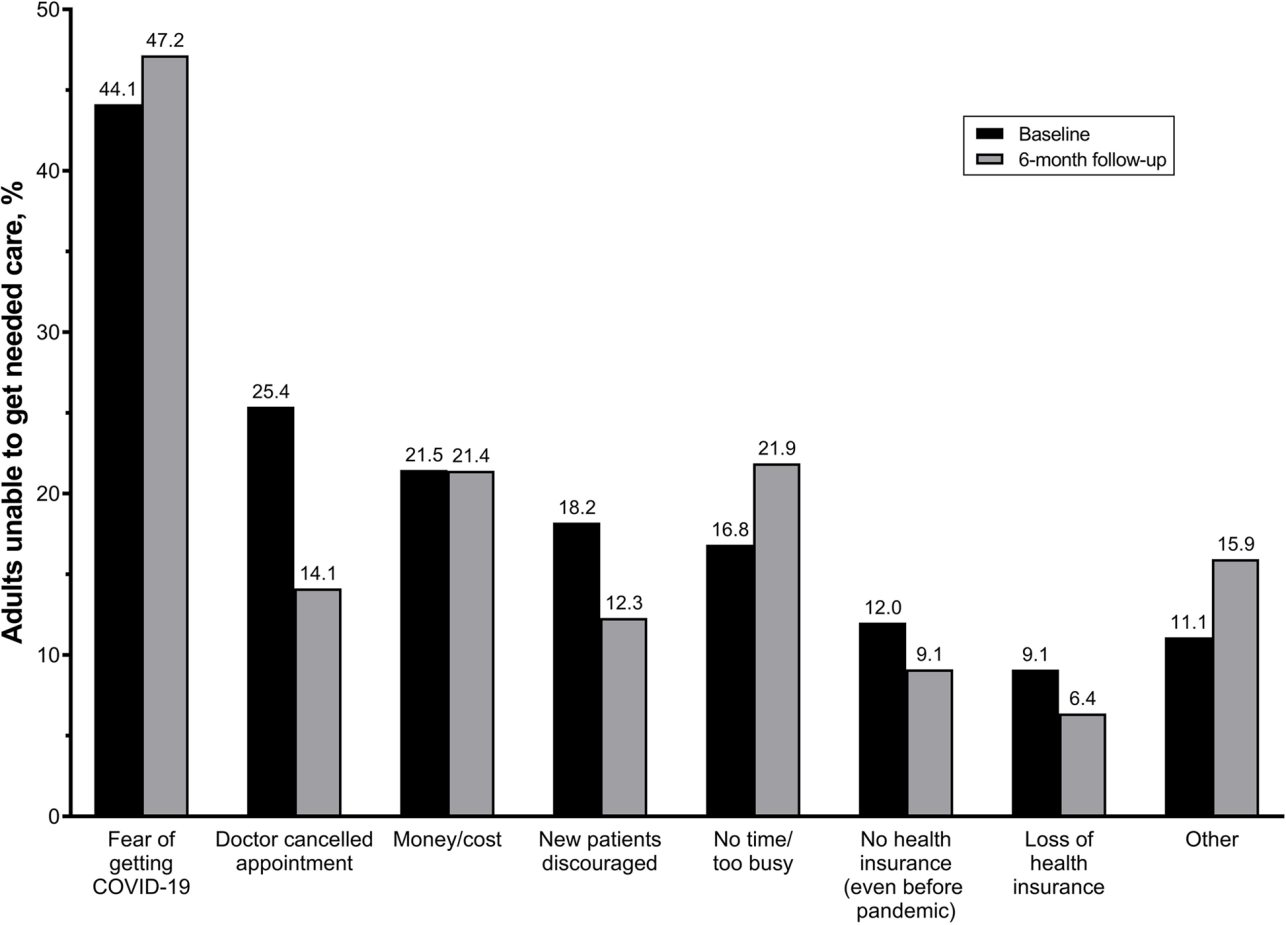
Reasons for not getting needed care among those who reported being unable to get needed care at baseline and 6-month follow-up. Baseline estimates were weighted to be nationally representative within each racial/ethnic group

At both baseline and 6-month follow-up, the most commonly reported reason for not receiving needed care was fear of getting COVID-19 (baseline: 44.1%, follow-up: 47.2%), Fig. 3. At baseline, the next most common reasons for being unable to receive care were doctors cancelling appointments (25.4%) and no time/too busy (21.9%), Fig. 3. This held true across all racial-ethnic groups and chronic condition status with the exception of American Indian/Alaska Native and multiracial adults with one or more chronic conditions who were both more likely to report a doctor cancelled their appointment than fear of COVID-19 (American Indian/Alaska Native: 41.3% vs. 40.4%; Multiracial: 49.3% vs. 35.6%), Supplemental Fig. 4A. Among individuals with one or more chronic conditions, money/cost was most often reported among Asian participants (39.4%), and doctor discouraged new appointments was most frequently reported among Native Hawaiian/Pacific Islander (50.4%) and multiracial (47.6%) adults, Supplemental Fig. 4. Similar trends in reasons for being unable to get needed care at follow-up were largely observed, Supplemental Fig. 5.

## Discussion

Using data from a nationally representative, diverse cohort of adults living in the U.S., we found that 20% of adults reported being unable to get needed care in early 2021 (December 2020-January 2021) and 22.7% reported being unable to get needed care in late summer 2021 (August–September 2021). Routine care/checkup and chronic care management were the most common types of missed care among adults with and without chronic conditions. Adults who were younger, uninsured, and reported fair/poor self-reported physical health were more likely to have experienced an inability to get needed care regardless of chronic condition status at baseline and follow-up. Fear of getting COVID-19 was the most frequently reported reason for being unable to get needed care, with almost half of all adults who were unable to get needed care reporting this as a reason.

Consistent with other studies conducted in the U.S. [2, 24, 25], we found that an inability to get needed care was more common among certain racial-ethnic groups, younger adults, uninsured adults, and adults with poorer self-reported physical health. Similar disparities have been reported in Brazil [26], the Netherlands [27], and Switzerland [28]. Before the COVID-19 pandemic, disparities in access to health care and health outcomes existed for vulnerable groups with those experiencing linguistic, socioeconomic, and geographic barriers being the most likely to delay and/or not get needed care [29, 30]. Such a sustained inability to get needed care among vulnerable populations during the pandemic could result in greater inequities in healthcare access and outcomes in the U.S [31]. This study adds to the existing research on health care delays in the U.S. during the pandemic, which has focused on older adults and adults with disabilities [5, 25, 32, 33], by including all adults regardless of age or disability, a broad range of racial-ethnic groups, and distinguishing the experiences of those with and without chronic conditions.

In our study, routine care was the most frequently reported type of care that individuals indicated they were unable to receive. Similarly, other U.S. based studies have found general doctor or specialist visits and scheduled preventative care were among the most forgone types of care [4, 32]. Routine care is critical for disease screenings, vaccination, and detecting disease early to prevent further deterioration of health [34]. Previous U.S.-based studies have shown that racial-ethnic minorities and uninsured individuals are less likely to have established usual sources of care [35, 36] and have less access to primary care providers [37]. These disparities have been attributed to language barriers and socioeconomic conditions that make accessing and utilizing health care less feasible [36, 38]. Disparities in the prevalence of chronic conditions across racial-ethnic groups and other vulnerable populations in the U.S. have also been well reported [39–41]. Given that disruptions in chronic care management can have a major impact on health and quality of life [14], as well as worsen health disparities [17, 18, 42], public health policy to support continuity of care post-pandemic is likely needed in the U.S. and other countries that experienced substantial delays.

In the U.S., health insurance and the ability to afford health care are critical for accessing health care services. The major economic impacts of the pandemic had downstream effects on health coverage and the ability to pay for health care expenses due to large increases in unemployment, depletion of savings, and loss of employer-sponsored health insurance [43–45]. In our study, a large proportion of uninsured individuals (48.6%) with chronic conditions reported being unable to get needed care, at a higher prevalence than those with both private and public health insurance. Additionally, about one-fourth of adults with chronic conditions reported money/cost as the reason for not getting needed care. Individuals with chronic conditions often require regular interactions with their caregivers to monitor and mitigate progression of disease, and without health insurance the cost of these services is significantly higher and can substantially reduce access to care [46]. Prolonged and acute disruptions in care, particularly for those with chronic conditions, can be detrimental in both short-term and long-term outcomes [17]. Moving forward, efforts to reengage (and engage) patients in chronic care management, routine care, and preventive care will be critical to avoid poor health outcomes.

At baseline, fear of COVID-19 infection was the most frequently reported reason for not getting needed care. As individuals with chronic conditions and those with unmet health-related basic needs are particularly vulnerable to severe COVID-19 infection, clinical settings may have been viewed as high-risk locations during the peak of the pandemic [2, 5, 47]. In our study, 36% to 68% of adults from all major racial-ethnic groups indicated that fear of getting COVID-19 was one of the primary reasons for not getting needed care. Other studies in both the U.S. and abroad, have also found that worry about COVID-19 exposure was a major reason for delaying or forgoing health care, especially among individuals with chronic conditions who felt they were at high risk for COVID-19 morbidity and mortality [4, 32, 48, 49].

“Doctor cancelled appointment” was the second most reported reason for not getting needed care at baseline and was the top reason among American Indian/Alaska Native and multiracial adults. This study is the first to report the unique experiences of American Indian/Alaska Native and multiracial adults with health care in the U.S. during the COVID-19 pandemic. Populations utilizing or relying on the Indian Health Service (IHS) faced barriers to care even before the COVID-19 pandemic, including poor facility funding, a lack of pharmaceuticals, technology, and fewer than required health professionals during non-emergent times [50–52]. The COVID-19 pandemic put additional strain on healthcare systems [53] and as they continue to recover and rebuild service capacity, it is essential to reestablish preventive care screening and non-COVID related health care practices. Other studies in the U.S. have similarly found a large proportion of individuals reporting cancelled or postponed appointments, as well as cancellations being more common among individuals with medical conditions and racial-ethnic minorities [2, 54, 55].

This study is not without limitations. First, our follow-up sample size limited our ability to detect statistically significant differences in the frequency and reasons for being unable to receive needed care, and we were unable to generate nationally representative estimates through weighting. Our survey also had a relatively low initial response rate among YouGov panel members (20.0%). Second, there may have been heterogeneity in responses to the COVID-19 pandemic owing to differences in the extent of the pandemic and public health responses in different locales not captured in our survey. Third, we limited our questions about chronic conditions to those defined as increasing risk of severe COVID-19 illness, which did not include other chronic conditions that may require regular management, and likely misclassified some adults as not having a chronic condition.

Fourth, our survey item on inability to get needed care was asked before asking what type of care was delayed, therefore there may have been variation in the perception/definition of “needed health care” leading to potential underestimation. Our reasons for being unable to get needed care were also broad, and future studies looking into more specific reasons/mechanisms for skipped or delayed care are needed. For example, racial/ethnic minorities were more likely to be essential workers during the COVID-19 pandemic, which may have created additional challenges in accessing care while navigating work schedules and lack of sick leave to see a doctor [56], but we only asked participants if the reason they were unable to get needed care was because “no time/too busy”. Fifth, the CURB survey was only administered in English and Spanish (Latino participants only), which means non-English speaking adults and non-Spanish speaking Latinos were more likely to be excluded. This may have especially impacted our Asian adult cohort, as 31.9% of Asian adults nationally are estimated to have limited English proficiency (compared to 12.3% in our survey) [57]. The survey was also administered online, and individuals with limited internet access or familiarity with technology and limited literacy may have been less likely to participate. Finally, while our survey was designed to be representative of the major US racial-ethnic groups, stratified results for Asian, Latino, and Native Hawaiian/Pacific Islander adults by heritage/country of origin may not be representative and sample sizes were small in some groups.

The findings of this survey study suggest that as the U.S. recovers from the COVID-19 pandemic, we should continue to support efforts to fortify access to health care services and ensure that vulnerable populations are a priority. As routine care and chronic care management were the most common types of needed health care that were not obtained during the pandemic, it is imperative that outreach efforts are bolstered to reestablish adherence to recommended preventative screenings, chronic care management and monitoring, and establishment of a suitable usual source of care. Further, as the national emergency and public health emergency declarations related to the COVID-19 pandemic ended on May 11, 2023, it is imperative that affordability and accessibility of health care services be ensured especially for individuals and communities suffering the most severe impacts of the COVID-19 pandemic.

## Conclusions

Our study has described the prevalence of being unable to get needed health care, the types of health care, and the reasons during the COVID-19 pandemic among U.S. adults with and without chronic conditions. Sociodemographics associated with increased likelihood of an inability to get needed care are also reported. The consequences of postponed health care disproportionately faced by vulnerable populations are essential to address especially as we learn more about the deficiencies and shortcoming of the U.S. health care patchwork approaches to health care that have been taken during the COVID-19 pandemic. As we continue to experience the long-term ramifications of the pandemic and a rise in the spread of communicable diseases it is essential to fortify healthcare infrastructure to ensure care is accessible and received in a timely manner to prevent the development and progression of disease, even in the face of a global pandemic. We can take this new knowledge and work towards developing reasonable health care access plans that directly address disparities in the communities most affected by an inability to receive needed care to ensure preparedness for future pandemics and address the setbacks left behind by the COVID-19 pandemic. This includes establishing outreach programs for the reinforcement of the importance of preventive routine care such as routine cancer screening and informing at patients with chronic conditions on at home care/maintenance strategies for times when care is inaccessible, impractical, or high risk.

### Supplementary Information

**Additional file 1: Supplemental Figure 1.** Prevalence of being unable to get needed care, stratified by race/ethnicity and chronic condition status, at A) baseline and B) 6-month follow-up. Baseline results weighted to be nationally representative within each racial/ethnic group. **Supplemental Figure 2.** Prevalence of the four most common types of care participants were unable to receive at baseline, stratified by race/ethnicity *n*=1,099 (20%). among those with A) ≥1 chronic condition and B) no chronic conditions. All results weighted to be nationally representative within each racial/ethnic group. **Supplemental Figure 3.** Prevalence of the four most common types of care participants were unable to receive at follow-up among those with A) ≥1 chronic condition and B) no chronic conditions, stratified by race/ethnicity, *n*=439. Due to low response rates results are not weighted. **Supplemental Figure 4.** Prevalence of top four most common reasons for being unable to get needed care at baseline among participants with A) ≥1 chronic condition and B) no chronic conditions, stratified by race/ethnicity, *n*=1,099. All results weighted to be nationally representative within each racial/ethnic group. **Supplemental Figure 5.** Prevalence of top three most common reasons for being unable to get needed care at follow-up among participants with A) ≥1 chronic condition and B) no chronic conditions, stratified by race/ethnicity, *n*=439. All results weighted to be nationally representative within each racial/ethnic group. Due to low response rates results are not weighted.

## Data Availability

Data is available upon reasonable request. Contact Dr. Paula Strassle (paula.strassle@nih.gov) for access.

## Abbreviations

U.S.: United States
CMS: Centers for Medicare and Medicaid Services
CDC: Centers of Disease Control and Prevention
CURB: COVID-19’s Unequal Racial Burden
COPD: Chronic obstructive pulmonary disease
GED: Tests of General Educational Development
IHS: Indian Health Service
SD: Standard deviation
CI: Confidence interval
aPR: Adjusted prevalence ratio
