# Supplementary material for: Inability to get needed health care during the COVID-19 pandemic among a nationally representative, diverse population of U.S. adults with and without chronic conditions
Source: BMC Public Health. 2023 Sep 26;23:1868. doi: 10.1186/s12889-023-16746-w (PMC10523792; doi:10.1186/s12889-023-16746-w)

**Supplemental Figure 1.** Prevalence of being unable to get needed care, stratified by race/ethnicity and chronic condition status, at A) baseline and B) 6-month follow-up. Baseline results weighted to be nationally representative within each racial/ethnic group.


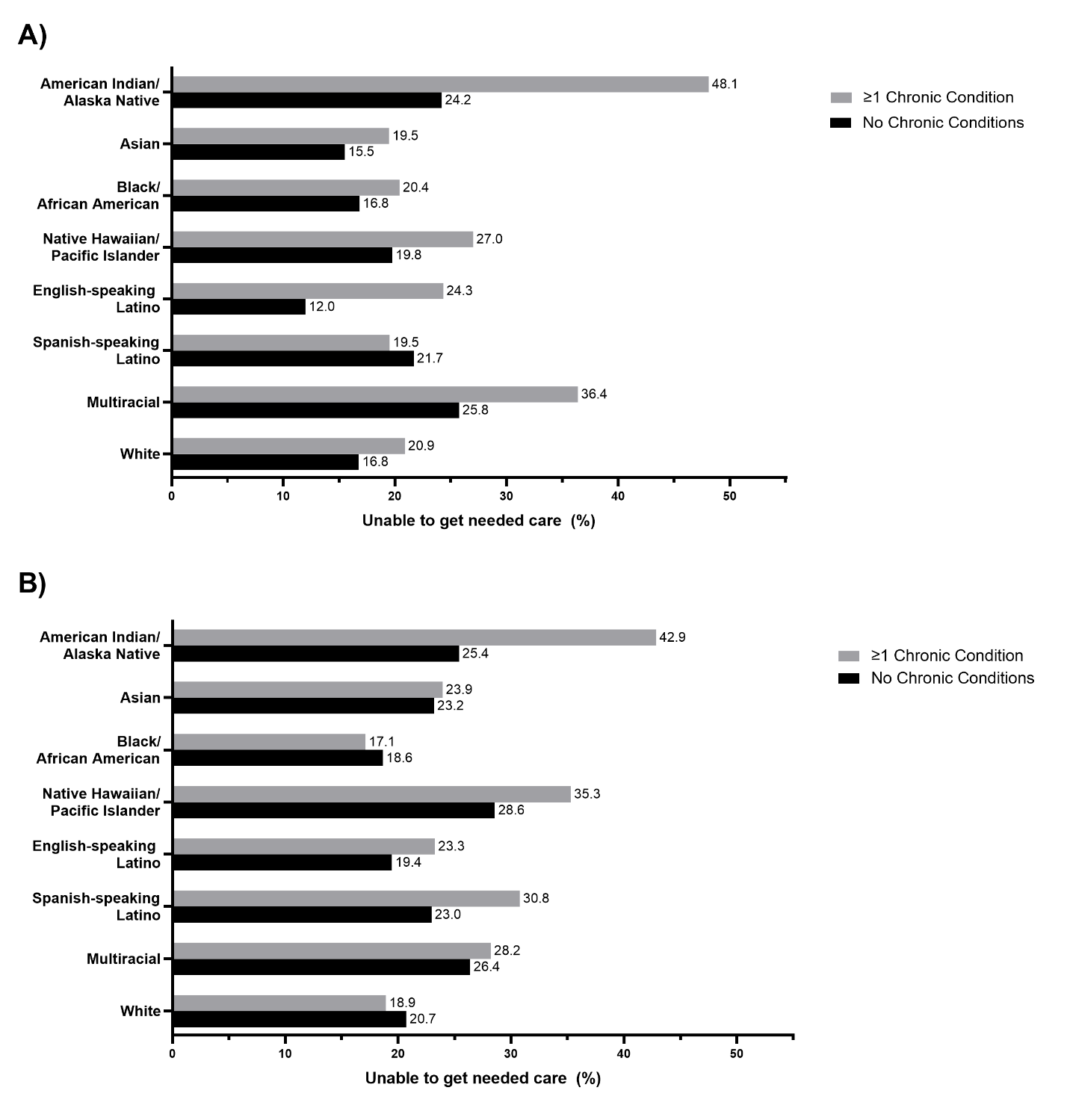


**Supplemental Figure 2.** Prevalence of the four most common types of care participants were unable to receive at baseline, stratified by race/ethnicity n=1,099 (20%). among those with A) ≥1 chronic condition and B) no chronic conditions. All results weighted to be nationally representative within each racial/ethnic group.


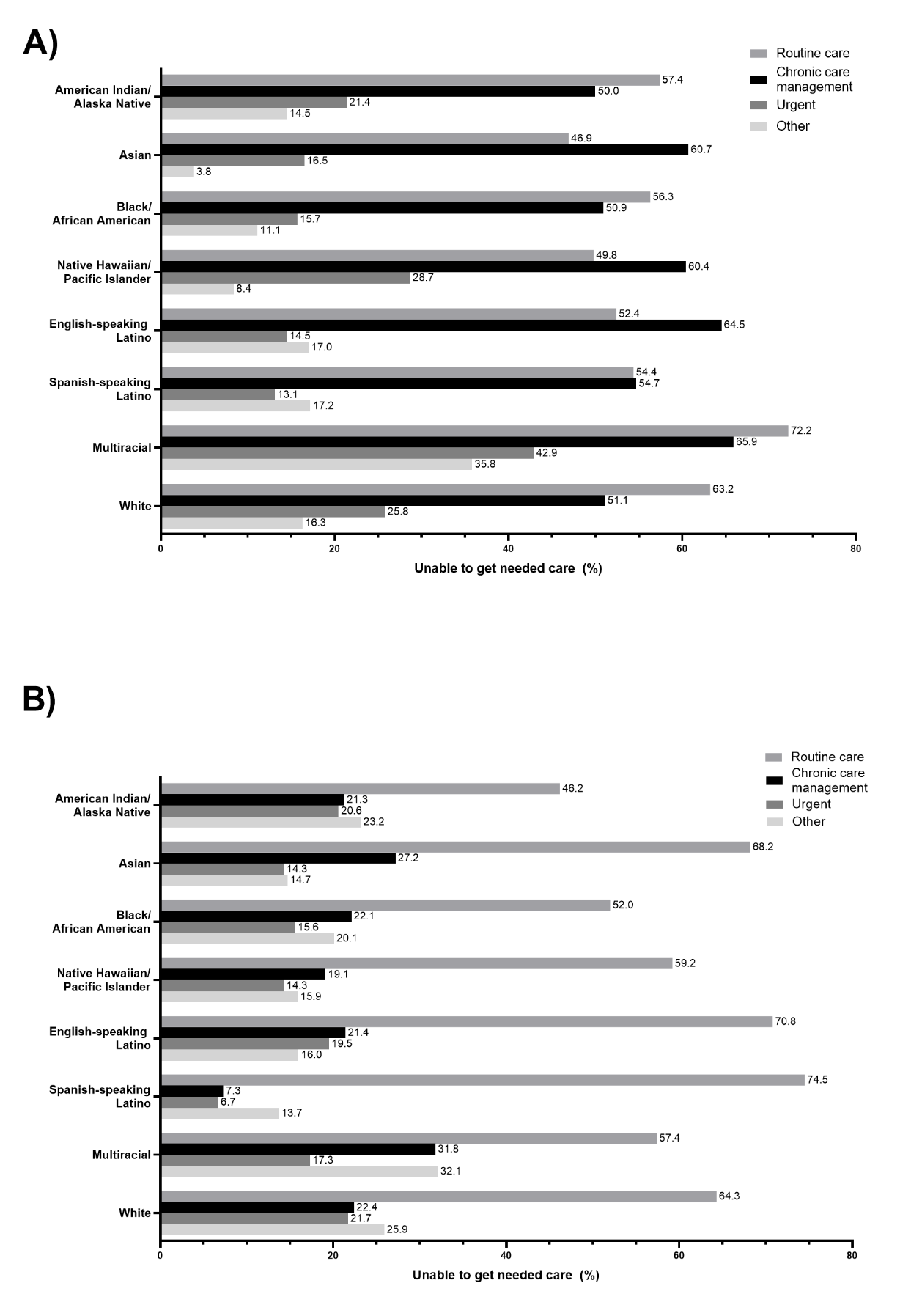


**Supplemental Figure 3.** Prevalence of the four most common types of care participants were unable to receive at follow-up among those with A) ≥1 chronic condition and B) no chronic conditions, stratified by race/ethnicity, n=439. Due to low response rates results are not weighted.


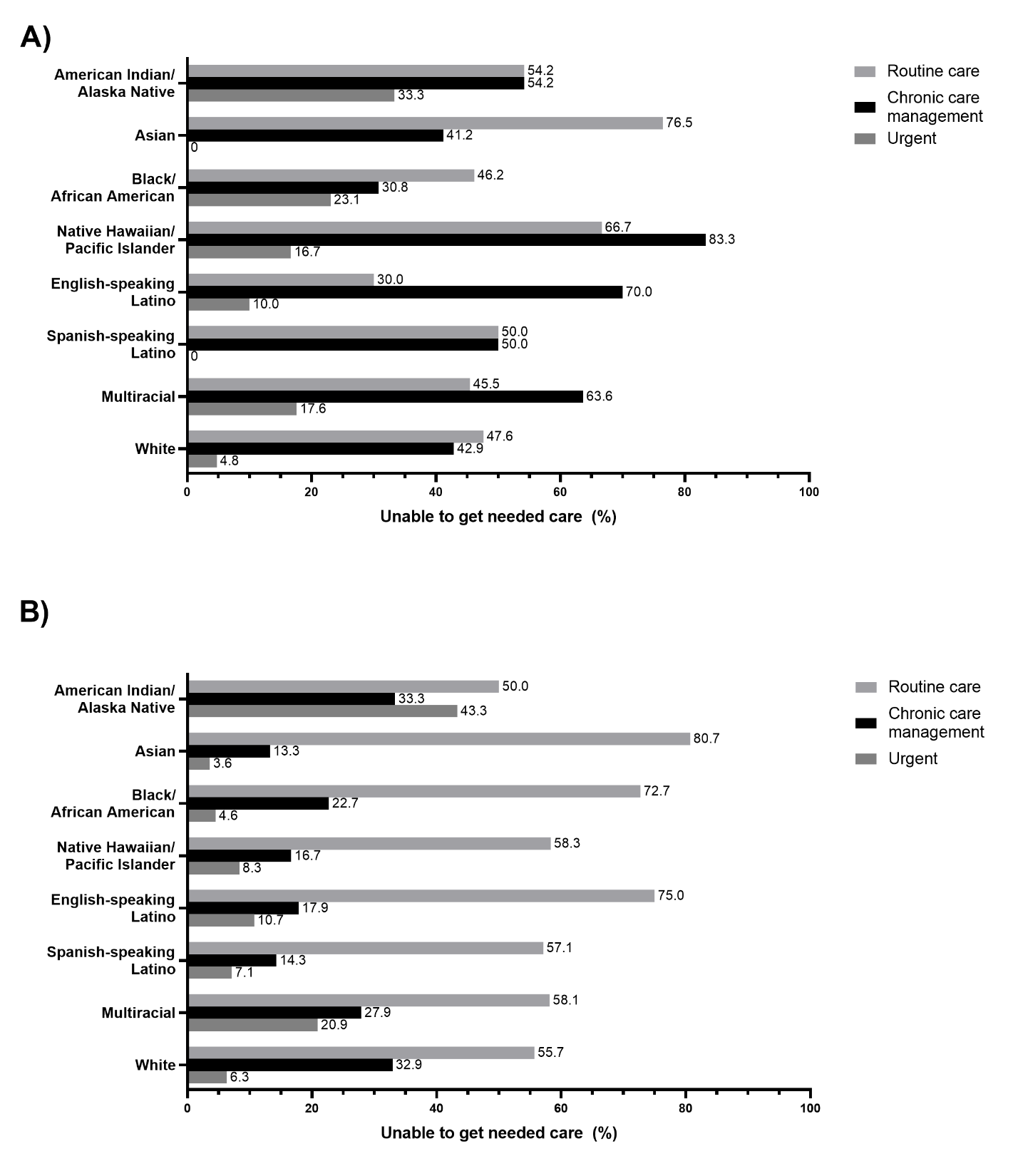


**Supplemental Figure 4.** Prevalence of top four most common reasons for being unable to get needed care at baseline among participants with A) ≥1 chronic condition and B) no chronic conditions, stratified by race/ethnicity, n=1,099. All results weighted to be nationally representative within each racial/ethnic group.


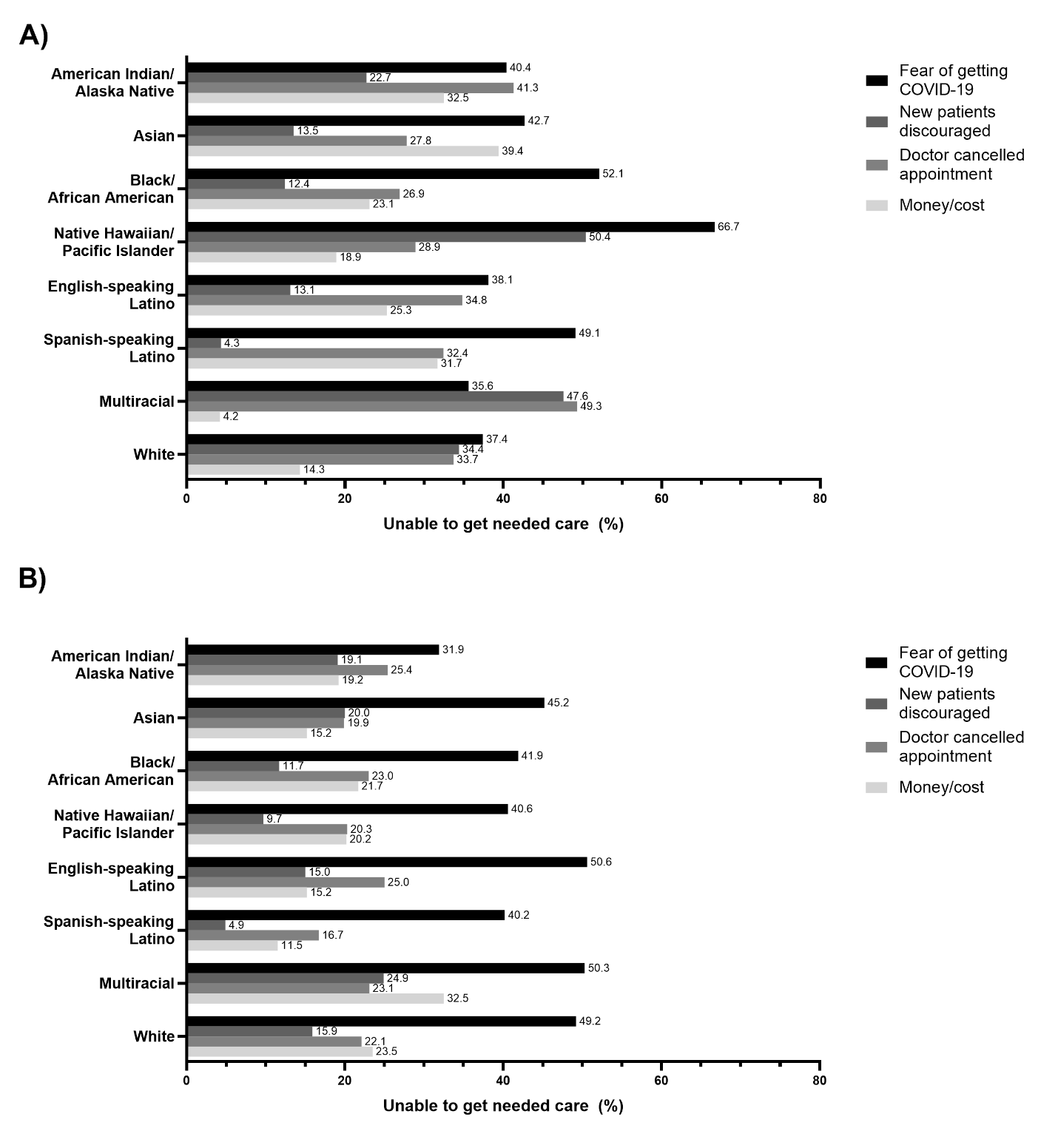


**Supplemental Figure 5.** Prevalence of top three most common reasons for being unable to get needed care at follow-up among participants with A) ≥1 chronic condition and B) no chronic conditions, stratified by race/ethnicity, n=439. All results weighted to be nationally representative within each racial/ethnic group. Due to low response rates results are not weighted.


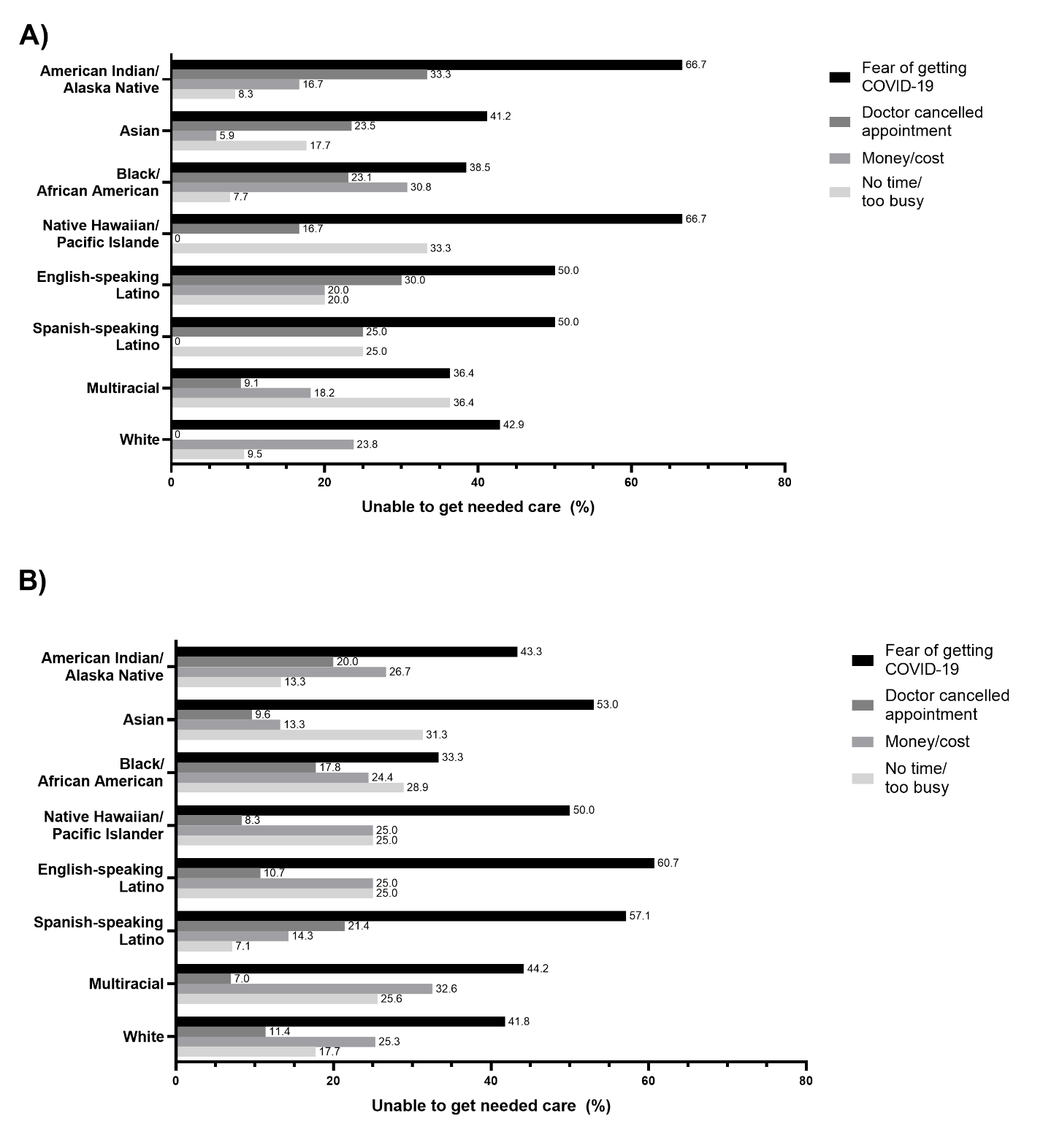

Supplement: Supplementary file 1 — Additional file 1: Supplemental Figure 1. Prevalence of being unable to get needed care, stratified by race/ethnicity and chronic condition status, at A) baseline and B) 6-month follow-up. Baseline results weighted to be nationally representative within each racial/ethnic group. Supplemental Figure 2. Prevalence of the four most common types of care participants were unable to receive at baseline, stratified by race/ethnicity n=1,099 (20%). among those with A) ≥1 chronic condition and B) no chronic conditions. All results weighted to be nationally representative within each racial/ethnic group. Supplemental Figure 3. Prevalence of the four most common types of care participants were unable to receive at follow-up among those with A) ≥1 chronic condition and B) no chronic conditions, stratified by race/ethnicity, n=439. Due to low response rates results are not weighted. Supplemental Figure 4. Prevalence of top four most common reasons for being unable to get needed care at baseline among participants with A) ≥1 chronic condition and B) no chronic conditions, stratified by race/ethnicity, n=1,099. All results weighted to be nationally representative within each racial/ethnic group. Supplemental Figure 5. Prevalence of top three most common reasons for being unable to get needed care at follow-up among participants with A) ≥1 chronic condition and B) no chronic conditions, stratified by race/ethnicity, n=439. All results weighted to be nationally representative within each racial/ethnic group. Due to low response rates results are not weighted. [file 12889_2023_16746_MOESM1_ESM.docx]
